# AI-Based Soft Module for Safe Human–Robot Interaction towards 4D Printing

**DOI:** 10.3390/polym14163302

**Published:** 2022-08-13

**Authors:** Ali Zolfagharian, Mohammad Reza Khosravani, Hoang Duong Vu, Minh Khoi Nguyen, Abbas Z. Kouzani, Mahdi Bodaghi

**Affiliations:** 1 School of Engineering, Deakin University, Geelong, VIC 3216, Australia; 2 Chair of Product Development, University of Siegen, Paul-Bonatz-Str. 9–11, 57068 Siegen, Germany; 3 Department of Engineering, School of Science and Technology, Nottingham Trent University, Nottingham NG11 8NS, UK

**Keywords:** 3D printing, 4D printing, silicon, soft materials, human–robot, AI

## Abstract

Soft robotic modules have potential use for therapeutic and educational purposes. To do so, they need to be safe, soft, smart, and customizable to serve individuals’ different preferences and personalities. A safe modular robotic product made of soft materials, particularly silicon, programmed by artificial intelligence algorithms and developed via additive manufacturing would be promising. This study focuses on the safe tactile interaction between humans and robots by means of soft material characteristics for translating physical communication to auditory. The embedded vibratory sensors used to stimulate touch senses transmitted through soft materials are presented. The soft module was developed and verified successfully to react to three different patterns of human–robot contact, particularly users’ touches, and then communicate the type of contact with sound. The study develops and verifies a model that can classify different tactile gestures via machine learning algorithms for safe human–robot physical interaction. The system accurately recognizes the gestures and shapes of three-dimensional (3D) printed soft modules. The gestures used for the experiment are the three most common, including slapping, squeezing, and tickling. The model builds on the concept of how safe human–robot physical interactions could help with cognitive and behavioral communication. In this context, the ability to measure, classify, and reflect the behavior of soft materials in robotic modules represents a prerequisite for endowing robotic materials in additive manufacturing for safe interaction with humans.

## 1. Introduction

Rapid recent progress in soft robotics has enabled more applications of robots related to human interactions and communications. Communication practices vary for different purposes of the robot. This could include verbal or non-verbal communication. Recently, physical touch communication between humans and robots has been deemed indispensable and much more popular compared to verbal delivery in almost every social robot [1]. For instance, vending machine interfaces, service robots in restaurants or shops, or even smart phones. One practical application of social robots in health and therapy is nursing robots in hospitals for physical and intellectual rehabilitation [2] or in children’s healthcare experiences [3]. A multisensory environment (MSE) module as part of a robot system can be defined as a space equipped with sensory materials that provide users with visual, auditory, and tactile stimulations, usually with the aim of offering stimulating or relaxing experiences to individuals with cognitive and behavioral impairments, including people with Profound Intellectual and Multiple Disabilities (PIMD) [4,5].

People with PIMD are characterized by severe cognitive and/or sensory disabilities, which lead to very intensive support needs. They are developmentally equivalent to a two-year-old and have other physical problems, such as cerebral vision impairment. Individuals with PIMD often have difficulties in communication, so caregivers must always be on the alert for their few communication signs, as they could interpret the individual’s messages. The MSE integrated with items that stimulate other senses is often utilized to trigger and elicit positive responses in interactions between people with PIMD and sometimes for therapeutic reasons [4,5]. These devices are typically equipped with aromatherapy, music, adjustable lighting, a projector, a rocking chair, bean bags, and weighted blankets [4,5]. However, many MSE products have restricted engagement capabilities and may elicit a limited set of responses. Therefore, more versatile MSE devices that interreact safely through means such as touch [6], sounds [7], lights [8,9] while generating visual, auditory, or tactile feedback are demanded.

This study presents a multisensory soft robotics module with potential use for educational and therapeutic purposes. The designs need to be sturdy, safe, modular, and adjustable, as each client has different preferences and personalities. This approach supports the more creative and personalized modules for an interactive communication module by giving the robotic body a voice in response to touch. Hence, a safe modular product made of soft material, particularly silicon, is introduced to translate excitement levels by detecting and classifying touch gestures to a scale of three different sounds. This study aims to recognize three different types of touch gestures based on categorizations of higher human intents through affective touch so that they reflect anger (slap), restful (tickle), and playful (squeeze) emotions.

### 1.1. Touch Gesture Sensing in Robots

Humans can use gestures to express attitudes and desires. To bring the computer closer to human life, this project focuses on applying touching gestures to human–robot interactions. This could help build a concept of how humans and robots can understand each other. The role that the sense of touch plays in emotional communication in humans and animals has been widely studied, finding relations to attachment, bonding, stress, and even memory [10,11]. Touch, as a common communicative gesture, is an important aspect of social interaction among humans. Several works focus on using touch as a valid modality to ascertain the user’s intention and claim evidence of the potency of touch as a powerful means of communicating emotions. Hence, touch is a natural way of interaction that can contribute to improving human–robot interaction (HRI) and promoting intelligent behavior in social robots.

In robotics, various types of sensors are used to classify touch gestures. A set of microphones is utilized for the swipe, tap, knock, and stomp gesture classifications with a high accuracy range of 91–99% [12]. However, background noise and the risk of interfering with the touch signal have been deemed the main drawbacks. In addition, the application of acoustic touch detection and classification is limited to small objects and solid materials, such as plastic or metal. Other tactile sensing technologies, such as resistive, capacitive, and optical transducers, could be considered in the MSE module [13]. For instance, optical touch gesture detection and classification of soft materials has not yet met widespread acceptance due to changing lighting conditions, posing classification reliability issues, and changing lighting conditions [14], and some of these approaches can only sense relatively simple motion and are difficult to embed inside deforming surfaces or elongational soft robots. In this work, a general sensing strategy using low-cost piezoelectric accelerometers and their efficiency in both touch gesture classification and various soft module sizes is demonstrated. The PVDF sensors were selected due to their flexibility, small size, and high sensitivity.

The Piezoelectric PVDF sensor can be used at a wide range of frequencies and is not bandlimited, so the amount of signal transferred is significant [15]. In addition, the lightweight and flexible properties of this sensor help lower the mechanical impedance [15]. The PVDF film sensor is also thin and flexible; hence, it is compliant with any type of surface. The sensor can easily be pasted on different materials. Compared to strain gauges, PVDF sensors are easier to install and can suit any size and geometry. This feature makes the PVDF film sensor stand out from the complicated installation process of a strain gauge.

### 1.2. Machine Learning Classification

The real-time classification and recognition of physical interactions, or proprioception, is a challenging problem for soft robots due to the many degrees of freedom (DOFs) and lack of available off-the-shelf sensors. In this work, low-cost piezoelectric sensors are used based on a data-driven machine learning (ML) strategy to classify proprioception input signals accurately in real time. Machine learning algorithms are also applied to determine the relationship between sensor data and shape-oriented parameters. To do so, the signals of different gestures on different soft modules with various shapes are acquired to create a dataset to train the model.

Different classifier algorithms have been used in ML for touch and gesture classification in human and robot interactions, including linear and quadratic support vector machines (SVM) [16], temporal decision trees (TDT) [17], and k-nearest neighbors (kNN) [18]. However, it was shown that, as per the type of sensors and the applications, the most suitable classification method could also be varied. Therefore, this study used different SVM classifier algorithms using the machine learning package in MATLAB as the classifying algorithm to classify different tactile gestures.

The rest of the paper is structured as follows: Section 2 describes the proposed system for touch detection and classification and details the hardware used in this work, as well as the fabrication of the soft robot module. Section 3 describes the feature collection process with the validation and testing of our dataset and discusses the results obtained. Finally, the conclusions of this work are presented in Section 4.

## 2. Methodology

**Figure 1:**
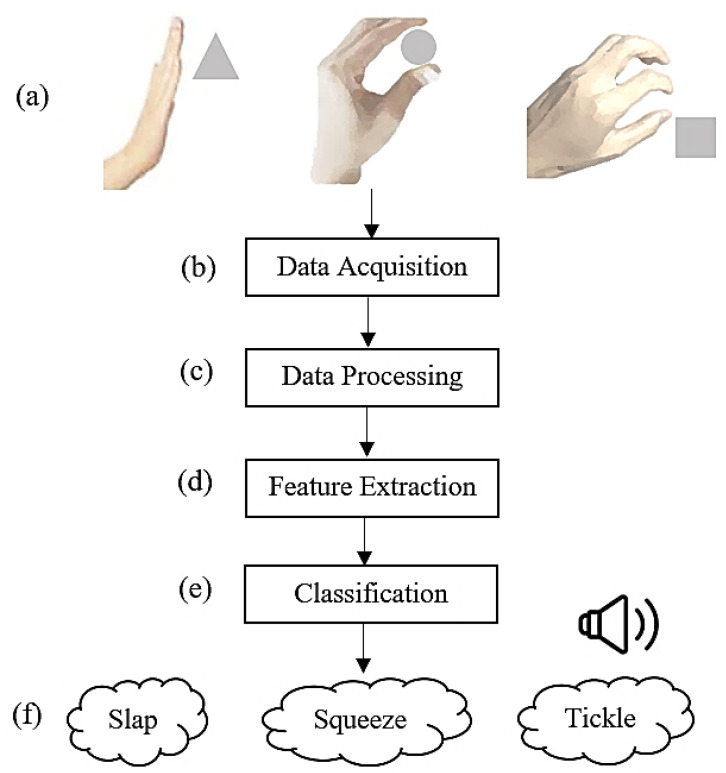
Touch gestures used for the classification in this study are Slap, Squeeze, and Tickle, as these are the most common actions that humans would perform in order to feel or trigger an object; (**a**) the touch is produced by the user; (**b**) the vibration is collected by the LDT0-028K PVDF sensor; (**c**) data processing and filtering; (**d**) feature extraction; (**e**) the classification using an ML algorithm; (**f**) the recognized gesture is verbally communicated.

The project’s aim is to design a physical touch communication robotics module that can recognize and differentiate the contact of a human finger and process the data using vibration sensors and a MATLAB machine-learning interface. The sound reaction command is implemented immediately after the prediction of the results so that the system calls the sound file when the gesture is identified. The different stages of the study are illustrated in Figure 1.

### 2.1. Fabrications, Hardware, and Data Acquisition

The main sensing module of the robot must be covered by a layer of soft material. The purpose of this action is to first insulate the electrical components from human touch, secondly transfer the proper vibration effect from human touches to the sensor, and thirdly help form the body of the soft robot. After several considerations on the material selection, silicon rubber was chosen as the most suitable material for the current soft robotics module project. This material was used to cover the piezoelectric sensor module. As the earlier study results showed, it has a more elastic characteristic than PLA materials for the touch vibration measurements [19] which are widely used materials for making soft robot bodies. Additionally, this material can be molded into any desired shape, is durable and resistant to mold and bacteria, and the electronic hardware or circuits can be embedded directly inside.

**Figure 2:**
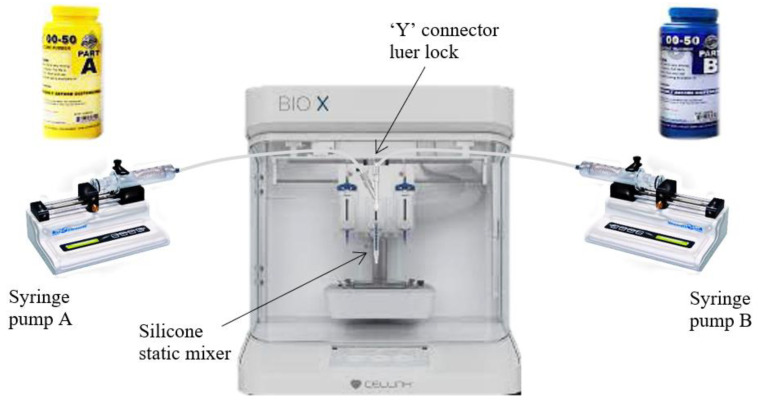
3D-printed mold and silicone modules.

**Figure 3:**
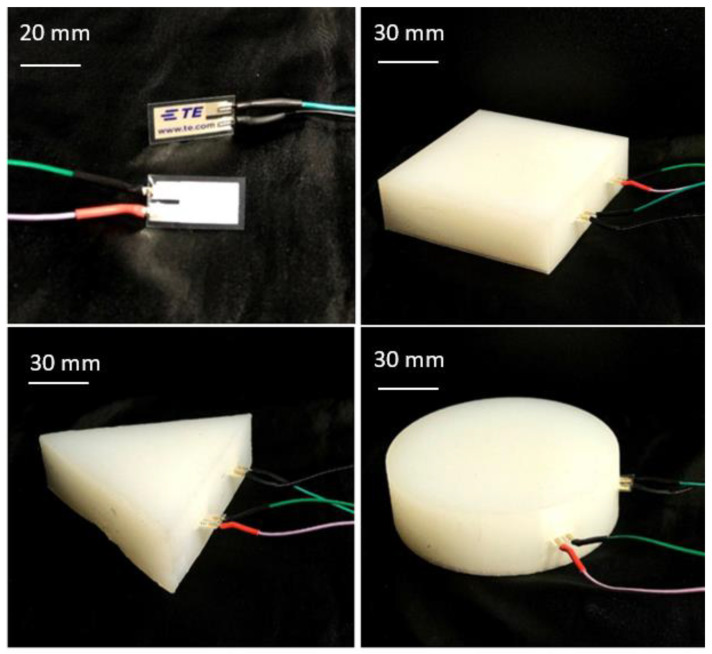
PVDF sensor with soldered wire and heat shrink; sensors embedded inside the silicon modules.

Ecoflex 00-50 was used in this work. The computer-aided design (CAD) of the soft robotic modules was developed using Autodesk Inventor and 3D-printed as per procedures used in [20]. The precursors of EcoflexTM 00-50, Part A and Part B (Smooth-On, Inc., Macungie, PA, USA), were used as the main material. For the silicone elastomer, 5 vol.% platinum silicone cure accelerator (Plat-Cat, Smooth-On, Macungie, PA, USA) and 1.1 vol.% silicone thickening agent (THI-VEX, Smooth-On, Macungie, PA, USA) were added to silicone part A. Furthermore, 20 g of Ecoflex-00-50-part A was loaded into syringe A, and 20 g of Ecoflex-00-50-part B was loaded into syringe B. Then, the syringes were loaded into the syringe pumps. The syringes were heated in the nozzle before being extruded, by a BIO X™—3D bioprinter—CELLINK, into a mold designated for holding the heater (Figure 2). The extruded ink in the mold was left at room temperature to be cured for 3 h according to the procedure used in [20,21]. Three shapes, including square, round, and triangle, were designed and developed, as shown in Figure 3. The purpose of making different shapes was to test the efficacy of ML algorithms on the recognition of different touches in different shapes of soft modules.

The PVDF sensors used were quite sensitive to vibration measurements. To reduce its sensitivity, the wires to the pins need to be soldered, and a heat shrink must be used to fasten the form-recovering process. Two sensors were embedded inside the silicon module so that the differences between the outputs of the sensor could be used as features to classify the types of touch. Additionally, the width of the testing pieces was 100 mm, and the width of the sensor was 13 mm. Hence, two sensors well spread in the testing piece would collect a wider range of vibration data and features from the touch. The embedded sensors are represented in Figure 3.

**Figure 4:**
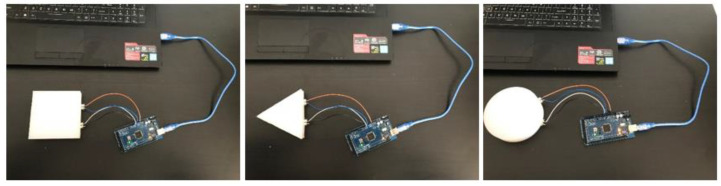
Set-up of silicon experiment modules.

**Figure 5:**
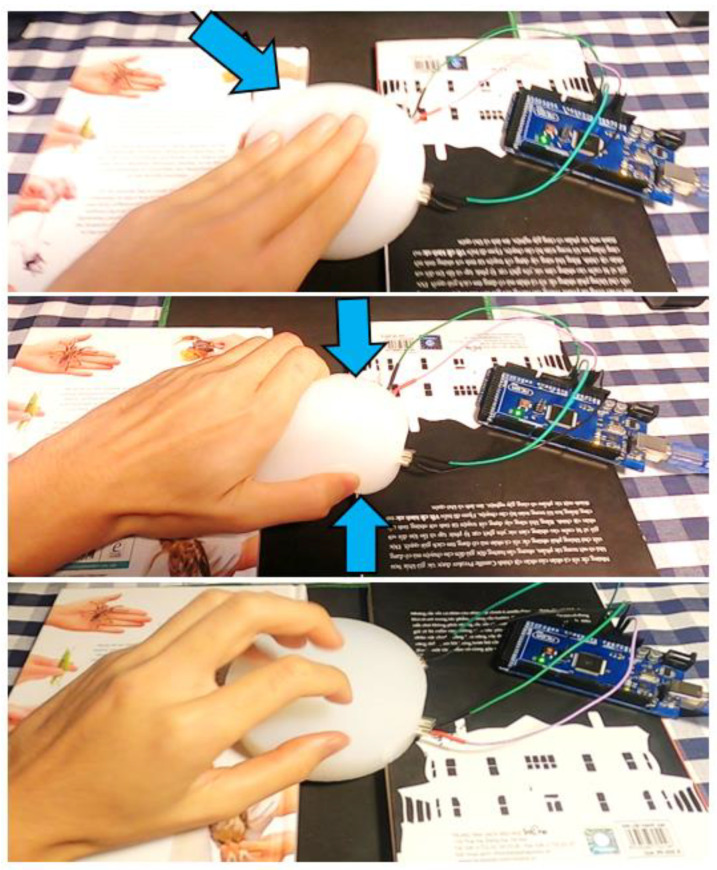
Applying different gestures from top to bottom; slapping with 4 fingers, squeezing with 5 fingers, and tickling with 4 fingers, respectively.

The micro-controller used in this project was the Arduino Mega2560. The microcontroller was connected to MATLAB on a laptop for interfacing and recording the signals (Figure 4). Three touch gestures were implemented to perform the experiment: slapping, squeezing, and tickling (Figure 5). These actions match the human emotions of boredom, anxiety, and anger, respectively. Thus, the module could realize what the user’s emotion is and, consequently, produce the appropriate soothing sound reaction. The experiment on each touch and shape was repeated 100 times to obtain enough data for the ML classification of touch gestures. This means that for three gestures, the total number of samples was 300. This was the number for only one shape. Because of the use of three shapes, the dataset of three gestures on three shapes contained approximately 900 samples.

### 2.2. Machine Learning Implementation for Gesture and Shape Classifications

Supervised learning is used to classify the touch gestures in this work. After being recorded, every signal sample is labeled with the correct type of gesture, along with the shape, called the correct output. All samples are summarized in a dataset. The classifying algorithm is trained with the dataset and makes predictions called predicted outputs. It will compare the correct outputs with the predicted outputs and modify the model accordingly. However, the classifying algorithm will not be trained with all samples in the received dataset. The acquired dataset is divided into two parts: one for training and one for validation. 60% of the dataset is used for training purposes, and the remaining 40% is for validation. For example, the set of tickling samples in a round shape had 100 samples. 60 samples were used to train the model, and 40 samples were left to validate its accuracy.

Five cases are set to test the accuracy of the model. The first case is to test the gestures in a round shape only. The second case is to compare the gestures in a round shape with those in a triangle. The third case is between the round and the square. The fourth happens with gestures in triangle and square shapes. The fifth is to combine all three shapes to see if the model can still classify with high accuracy.

The most important part of classification is extracting the features of the signals. These features are the keys to differentiating one type of touching from the others. Using a function called “extract” will assist in calculating the values for all features and summarizing them in a feature table (Supplementary Figure S1). The features that need to be extracted from the signals include duration, the aspect ratio of the signal between two sensors, the median, the mean absolute deviation, the maximum and minimum values, the velocity, and the correlation. After having a feature-extracting function, the next task is to run all the training samples through this function to acquire a feature table, which will later be used for training the model. The concept is also applied to all the gestures of the other shapes. After the extracting process, all the datastore feature tables for all the gestures were combined into one final feature table, which was used to train the model.

The MATLAB Classification Learning App was employed here. This application helps to apply all the classifying algorithms to the training data. The output of the app is a model that can be used in predicting the testing data. The training of the algorithm was executed in the Classification Learning App. The input is the features table, which was formed in the feature extraction stage. In the response section, three unique valuables are the three types of touching that need to be classified (Supplementary Video S1). Six types of SVM with different features, including Linear SVM, Quadratic SVM, Cubic SVM, Fine Gaussian SVM, Medium Gaussian SVM, and Coarse Gaussian SVM, were implemented, and the best results were used for validation (Supplementary Figure S2). The accuracy of the models was demonstrated by calculating the misclass rate. Finally, sketching a confusion matrix can present the number of gestures that are correctly predicted and the incorrect gestures, along with the types of misclassifications that the model misclassifies.

## 3. Results and Discussion

**Figure 6:**
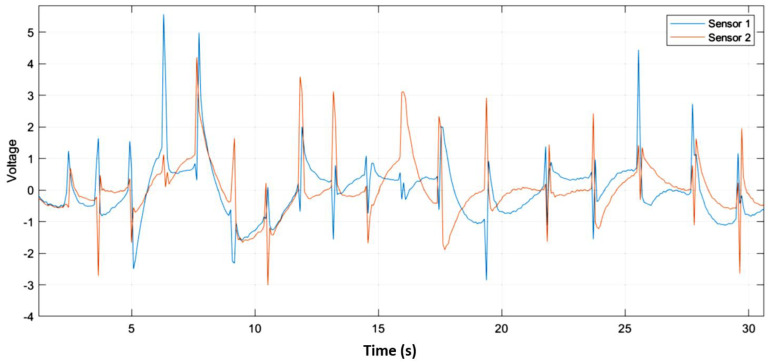

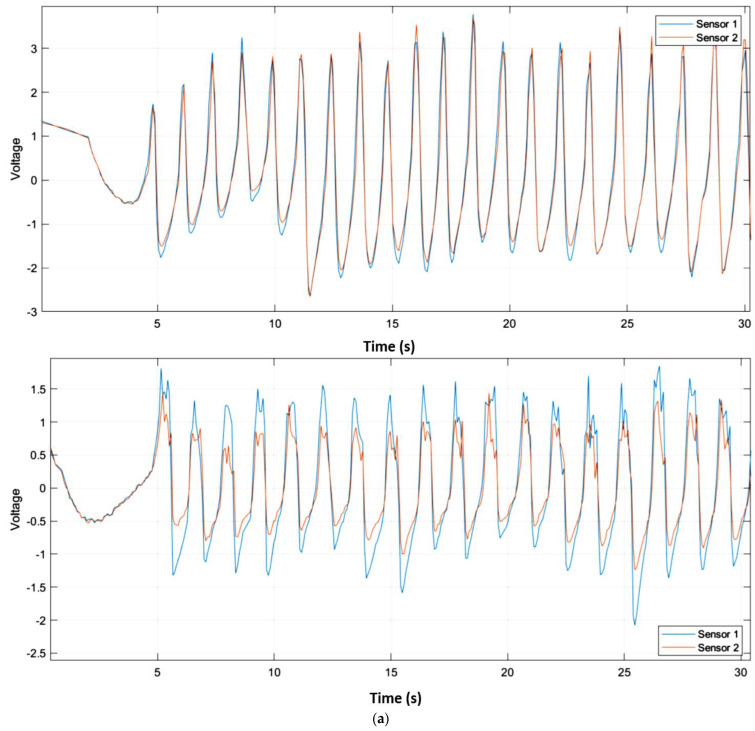

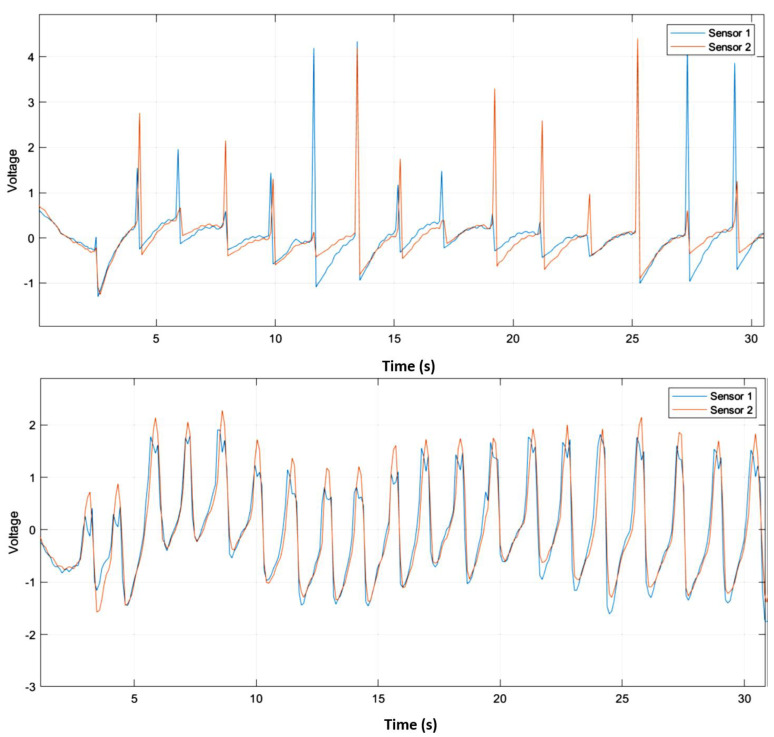

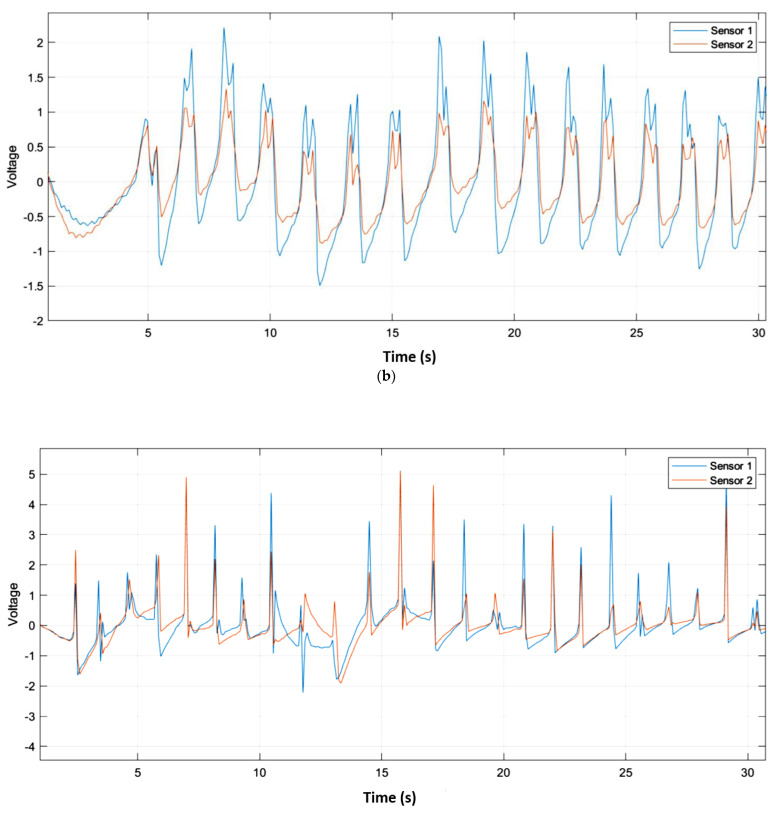

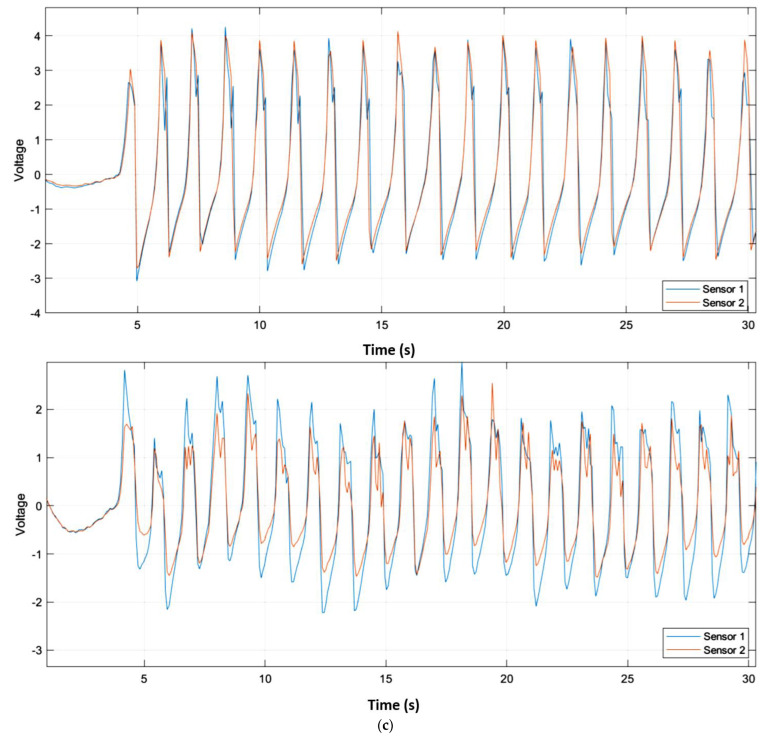
Data acquisition results of slapping, squeezing, and tickling from top to bottom, respectively, on (**a**) square, (**b**) round, and (**c**) triangle silicone modules.

A high-pass Chebyshev Type 2 filter was employed to process the signals. This will help feature extraction to go more easily and increase accuracy. The stop frequency is set to be 0.4 Hz, and the stop amplitude is 60 dB. The signals are time domain, and the achieved results in the first 30 s are shown in Figure 6. The results shown below was achieved by applying the gestures to the silicon module continuously. The red and blue features represent the output of sensor 1 and sensor 2 on the silicon module. Furthermore, the graphs showed that each gesture characteristic could be distinctly received by the sensors, producing a voltage linearly related to the magnitude of the touch force applied. This was deduced by the small but dense variation of the tickling graph, the large and widespread variation of the squeezing graph, and the sharp and widespread variation of the slapping graph.

The results are demonstrated in five cases. The first is a classification based solely on a round shape. Comparisons between round and triangle, round, and square, and triangle and square are the second, third, and fourth cases, respectively. The final case is to combine all three shapes into one classification session.

**Figure 7:**
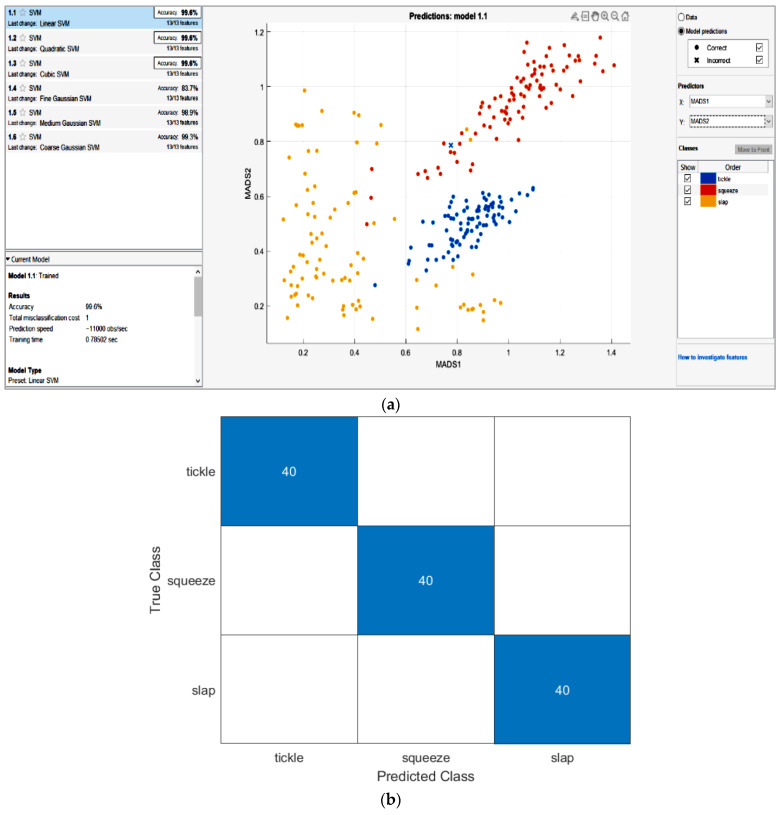
(**a**) The classification training algorithm plot and (**b**) Confusion Matrix of round module.

In the first case, the classification consists of three gestures in a round shape only. Therefore, the category of classifying variables includes tickling, squeezing, and slapping. The model was trained with all SVM algorithms, and three returned the highest accuracy of 99.6%, including Linear, Quadratic, and Cubic SVM, as shown in Figure 7a. The complexity of this case is the lowest because the model only needs to learn three types of gestures, and the amount of data is not large. Therefore, a linear SVM is sufficient to train the model in this case. The confusion matrix (Figure 7b) shows that all the testing samples were correctly classified, with no misclassified samples, in the round module. The accuracy percentage of the model in this case is 100%.

**Figure 8:**
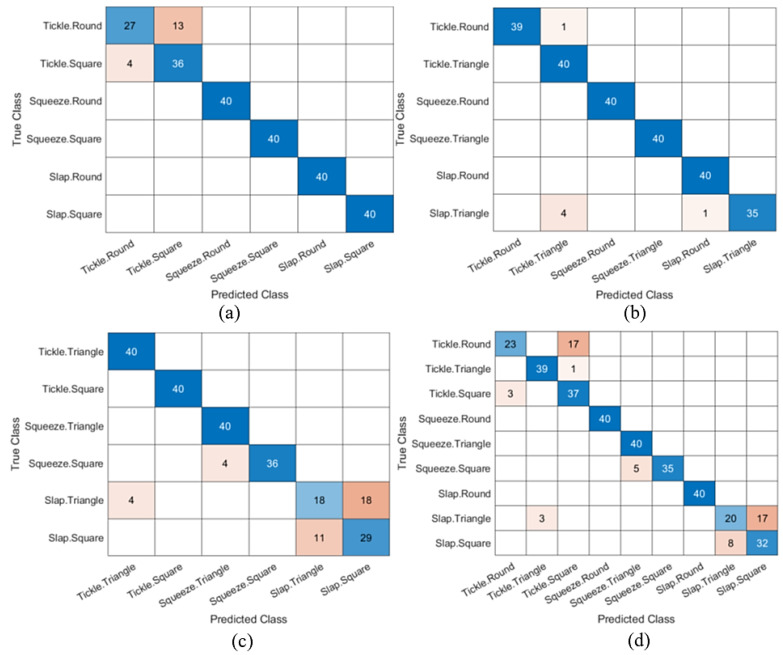
Confusion matrix of touch classification of different soft modules: (**a**) round vs square, (**b**) round vs triangle, (**c**) square vs triangle, and (**d**) all shapes.

In the second case, the model is meant to classify three types of touching on two shapes: round and square. This makes the number of classifying variables 6 instead. All SVM algorithms are also used to train the model. Fine Gaussian SVM returned the highest accuracy, up to 100%. However, the Fine Gaussian SVM model was not promising with the testing data. Therefore, the Cubic SVM model was used, reflecting the least amount of misclassification in the testing data. The confusion matrix had an accuracy of 92.92%, as shown in Figure 8a. The third case considered the classification of touch gestures on round and triangle, where again the Cubic SVM model was used to achieve the highest classification algorithms of 97.5%, as shown in the confusion matrix in Figure 8b. The scenario in the third study is similar to the two earlier cases, but the classification between square and triangle shapes and three touch gestures is shown in Figure 8c. However, the Cubic SVM model for this case had the lowest accuracy of 84.58%.

After testing with groups of two shapes, the final case is to combine all three shapes with the acceleration response of three touching gestures on each shape. The total number of classifying variables is 9. With a larger dataset, Quadratic SVM has shown the highest accuracy of 89.4%. Since the dataset for this case is the biggest, the cross-validation folds were set to be 5 folds. Cross-validation was applied to protect against overfitting by partitioning the dataset into folds and estimating the accuracy of each fold. As shown in the confusion matrix, the model could still classify with an acceptable accuracy of 85%, considering the larger pool of datasets.

As per the confusion matrix results (Figure 8d), the touch gestures of all the testing data could be successfully classified. However, in cases where the model classified three gestures as different shapes, the maximum accuracy dropped. In most cases, the samples that are misclassified are tickles and slaps. This could be explained by the fact that tickles and slaps were performed on the surface of the module. In contrast, the gesture of squeezing comes from two sides of the module. Moreover, round, triangle, and square are three different shapes, and the touchpoints of squeezing can be completely different. Thus, errors in classifying squeezes are less likely to occur.

The three touches on all shapes’ success rates are summarized in Table 1. It can be noticed in the tickling signals on round and square shapes that they are mostly similar. The tickling in a round shape and the tickling in a square shape have an equal number of peaks. Both happen within a duration of time and have a similar pattern ratio. This is also the cause of the errors in the two cases of round vs triangle and triangle vs square. The misclassed samples in round and triangle are the least among 3 cases that compare the gestures in pairs of shapes. The case that has the most misclassed samples is triangle vs square. The errors occur mainly in the classification of slapping samples of the two shapes. In brief, throughout the classification in all cases, the shapes of the module affected only the squeezing gesture. Meanwhile, tickling and slapping are less dependent on the shapes of the module because they are performed only on the surface, which is similar among all tested modules.

The tickling action on the shapes was almost the same. This was because this action was applied with the fingertips with light contact on the surface, which was the same for all shapes. The slapping gestures experienced the same, as this gesture occurred too quickly and powerfully, causing the graph to undergo a burst in voltage and then quickly go down swiftly. However, a minor difference can be seen right before the stabilized value. Squeezing gestures provided a slight difference between the triangle and the other two shapes. The logic behind this apparently came from the shape of the module. As stated above, the squeezing gesture was performed by placing pressure on the opposite sides of the module. Therefore, the width of the shapes contributed to the small variation, and the width of the triangle was the smallest of the three shapes, while the round and square shapes were all 100 mm in dimension.

## 4. Conclusions

A physically stimulated soft module for detecting and classifying human touch gestures, based on machine learning, was developed to help with stimulating or relaxing experiences for individuals with cognitive and behavioral impairments, including Profound Intellectual and Multiple Disabilities (PIMD) people and safe social robots, where the sound reaction resulting from the type of touch could be used to therapize the user’s emotional problems. The study provided knowledge of how a computer module was trained to recognize the three common touching gestures of humans, which are tickling, squeezing, and slapping, on different shaped soft modules, including round, triangle, and square.

First, Ecoflex 00-50 silicon-made soft modules were fabricated using 3D printing in different shapes. The touching gestures’ signals were then acquired on all three shapes by means of two PVDF accelerometers. Sufficient training and testing data were used for classification via the SVM algorithm in the machine learning package in MATLAB. The model has been trained with the data of three gestures from one shape, different pairs of shapes, and all three shapes together. The validating results were acceptable since the highest accuracy was 100% in differentiating the types of touch, and the lowest accuracy was 84.58%, classifying both shape and touch gestures. In detail, only the squeezing gesture is affected by the module’s forms. Tickling and slapping, on the other hand, are less affected by the shape of the module since they are conducted solely on the surface, which is consistent across all the evaluated modules. The thickness of the module critically affected the efficiency of the classifying process; the thinner the module, the noisier the data became.

The suggested module may be used by a wider population to inspire curiosity, reduce stress, and improve well-being via the ability to identify and categorize touch gestures, either as a stand-alone system or in conjunction with conventional touch sensing methods. The module should be operated wirelessly so that the user can bring it and put it anywhere. The design of the complete module should be more decorative and user-friendly, i.e., the color and shape of the module could be improved and have different options with all the electronic components hidden and 3D-printed. An inflatable silicone robot is a future direction. In addition, with the luxury of 4D printing, more adjustable and customizable modules are possible.

## Figures and Tables

**Table 1:** The three touches on all shape success rates.

Touch	Module	# Successes	# Failures	Success Rate (%)
Tickle	Round	23	17	57.5
Tickle	Triangle	39	1	97.5
Tickle	Square	37	3	92.5
Squeeze	Round	40	0	100
Squeeze	Triangle	40	0	100
Squeeze	Square	35	5	87.5
Slap	Round	40	0	100
Slap	Triangle	20	20	50
Slap	Square	32	8	80

## Data Availability

The datasets generated during and/or analysed during the current study are available from the corresponding author on reasonable request.

## Supplementary Materials

The following supporting information can be downloaded at: https://www.mdpi.com/article/10.3390/polym14163302/s1, Figure S1: Features Extraction function.; Figure S2: Training algorithms and plot snapshots on (a) round vs square, (b) round vs triangle, (c) square vs triangle, (d) all three shapes, from top to bottom respectively; Video S1: Demonstrations of touches.
