# Supplementary material for: AI-Based Soft Module for Safe Human–Robot Interaction towards 4D Printing
Source: Polymers (Basel). 2022 Aug 13;14(16):3302. doi: 10.3390/polym14163302 (PMC9416509; doi:10.3390/polym14163302)
Supplement: Supplementary file 1 [file polymers-14-03302-s001.zip › Supplementary Materials.pdf]

Supplementary Materials

A Safe Human-Robot Physical Interaction-Communication Module with Clinical Potentials

Ali Zolfagharian[1*], Mohammad Reza Khosravani[2], Hoang Duong Vu[1], Minh Khoi Nguyen[1], Abbas Z Kouzani[1], Mahdi Bodaghi[3*]

[1]School of Engineering, Deakin University, Geelong, Victoria 3216 Australia

[2]Chair of Product Development, University of Siegen, Paul-Bonatz-Str. 9-11, 57068 Siegen, Germany

[3]Department of Engineering, School of Science and Technology, Nottingham Trent University, Nottingham NG11 8NS, UK

* Author for correspondence: a.zolfagharian@deakin.edu.au

This file includes:

Supplementary Text

Figure S1: Features Extraction function.

Figure S2: Training algorithms and plot snapshots on (a) round vs square, (b) round vs triangle, (c) square vs triangle, (d) all three shapes, from top to bottom respectively

Video S1: Demonstrations of touches

```
function feat = extract(touch)
    %Duration
    duration = touch.Time(end) - touch.Time(1);

    %Aspect Ratio
    aratio = range(touch.Sensor1)/range(touch.Sensor2);

    %Median
    medS1 = median(touch.Sensor1,"omitnan");
    medS2 = median(touch.Sensor2,"omitnan");

    %Mean absolute deviation(MAD)
    devS1 = mad(touch.Sensor1);
    devS2 = mad(touch.Sensor2);

    %Local max/mins
    idxmin1 = islocalmin(touch.Sensor1);
    numminS1 = nnz(idxmin1);
    idxmax1 = islocalmax(touch.Sensor1);
    nummaxS1 = nnz(idxmax1);

    idxmin2 = islocalmin(touch.Sensor2);
    numminS2 = nnz(idxmin2);
    idxmax2 = islocalmax(touch.Sensor2);
    nummaxS2 = nnz(idxmax2);

    %Velocity
    dT = diff(touch.Time);
    dX1dT = diff(touch.Sensor1)./dT;
    dX2dT = diff(touch.Sensor2)./dT;
    avgdS1 = mean(dX1dT,"omitnan");
    avgdS2 = mean(dX2dT,"omitnan");

    %Correlation
    corrS1S2 = corr(touch.Sensor1,touch.Sensor2,"rows","complete");
```

Figure S1. Features Extraction function.

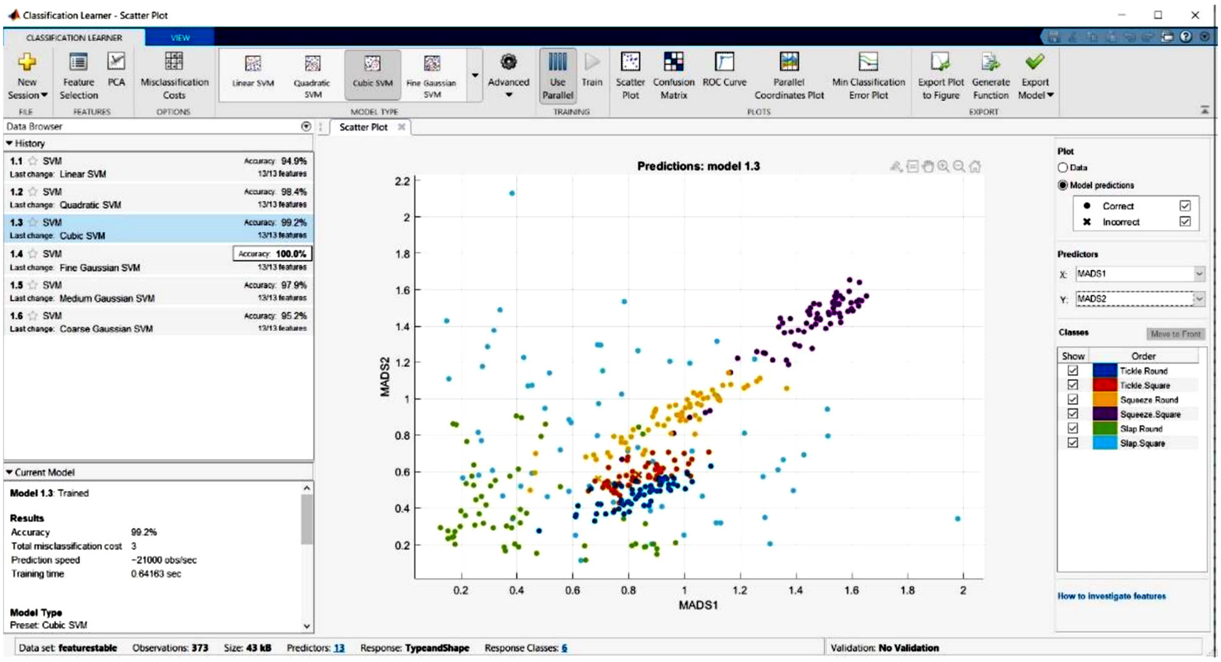

(a)

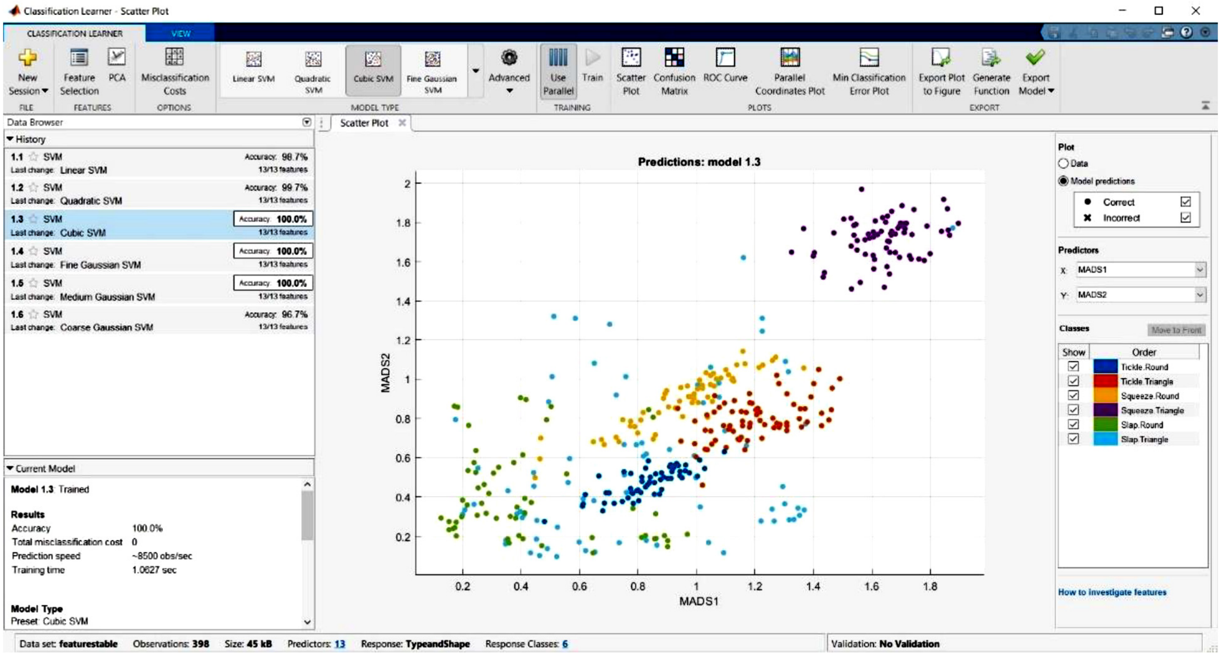
Classification Learner - Scatter Plot
Predictions: model 1.3
MADS1
MADS2
Model 1.3 Trained
Accuracy 100.0%
Total misclassification cost 0
Prediction speed ~8500 obs/sec
Training time 1.0627 sec
Model Type
Preset: Cubic SVM
Tickle Round
Tickle Triangle
Squeeze Round
Squeeze Triangle
Slap Round
Slap Triangle
Data set: featurestable Observations: 398 Size: 45 kB Predictors: 13 Response: TypeandShape Response Classes: 6
Validation: No Validation

(b)

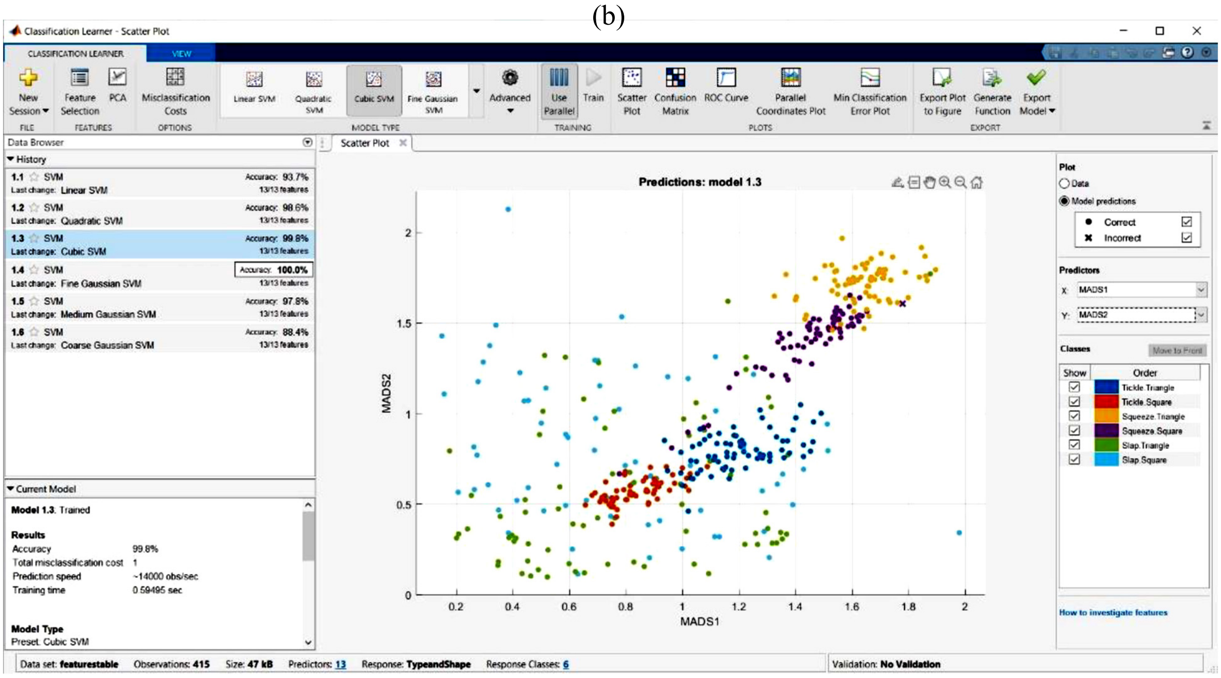
Classification Learner - Scatter Plot
Predictions: model 1.3
MADS1
MADS2
Model 1.3 Trained
Accuracy 99.8%
Total misclassification cost 1
Prediction speed ~14000 obs/sec
Training time 0.59495 sec
Model Type
Preset: Cubic SVM
Tickle Triangle
Tickle Square
Squeeze Triangle
Squeeze Square
Slap Triangle
Slap Square
Data set: featurestable Observations: 415 Size: 47 kB Predictors: 13 Response: TypeandShape Response Classes: 6
Validation: No Validation

(c)

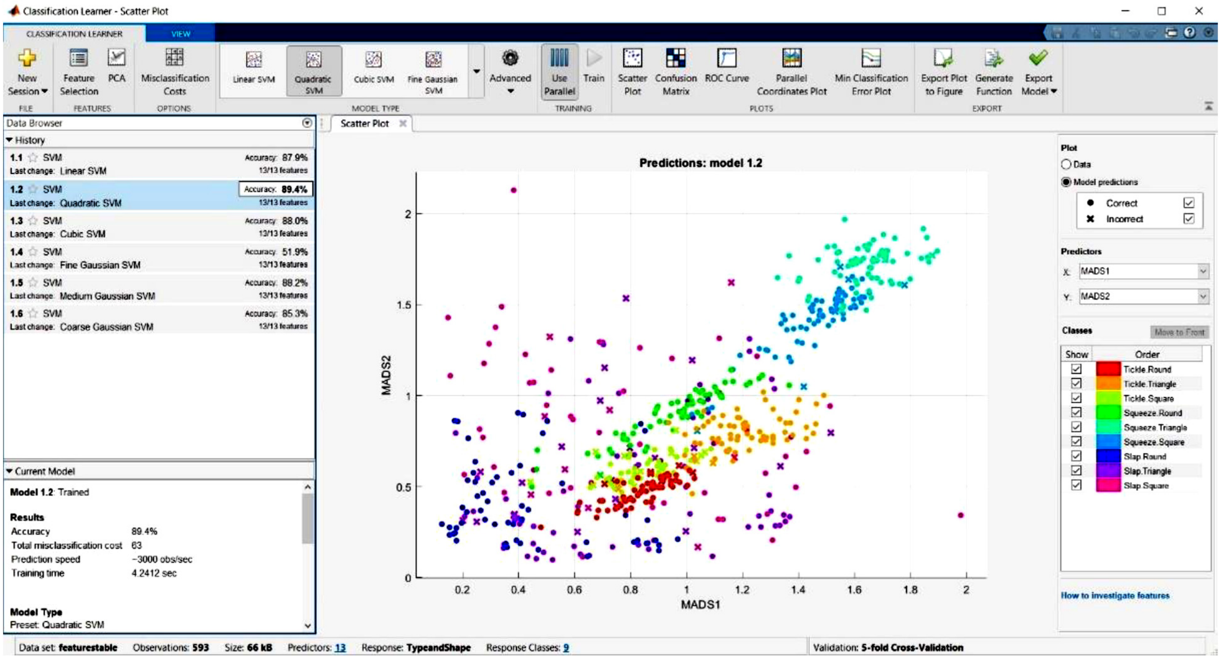

(d)

Figure S2. Training algorithms and plot snapshots on (**a**) round vs square, (**b**) round vs triangle, (**c**) square vs triangle, (**d**) all three shapes, from top to bottom respectively.
